# Assessing the Use of Left Atrial Strain Before and After Treadmill Exercise Stress Echocardiography

**DOI:** 10.1111/echo.70253

**Published:** 2025-08-01

**Authors:** Benjamin T. Fitzgerald, Jonathan Chan, Alfred K. Lam, Gregory M. Scalia

**Affiliations:** ^1^ Advara HeartCare Brisbane Australia; ^2^ Griffith University Gold Coast Australia; ^3^ The Wesley Hospital Brisbane Australia; ^4^ The Prince Charles Hospital Chermside Australia; ^5^ University of Queensland Brisbane Australia

**Keywords:** diastolic function, echocardiography, left atrial strain, stress testing

## Abstract

**Aims:**

Left atrial strain (LAS) is a novel speckle‐tracking technique used in the assessment of left atrial function. It has been shown to be a marker for adverse cardiac events (heart failure, myocardial ischemia, and atrial fibrillation). This study aimed to assess the role of LAS in assessing risk for diastolic dysfunction and myocardial ischemia in stress echocardiography (SE) before and after treadmill exercise.

**Methods and Results:**

Patients undergoing routine Bruce protocol SE had LAS measurements (reservoir, conduit, and contractile strain) assessed before and after maximal treadmill exertion. Changes in LAS were measured and compared based on the result of the SE. Consecutive SE (*n* = 420) were analyzed (mean age 62 ± 5 years, 37% female). LAS was able to be estimated before and after stress in 367 patients, including 313 *Normal SE*, 32 *Ischemic SE*, and 22 *Abnormal DST*. All LAS measurements (reservoir, conduit, and contractile strain) increased significantly in *Normal SE*. The LAS measurements did not significantly change for patients with *Ischemic SE* and *Abnormal DST*. Using a receiver operator curve analysis and linear regression, apical 4‐chamber reservoir LAS was found to be the best differentiator between *Normal SE* and abnormal tests (*Ischemic SE* and *Abnormal DST)*.

**Conclusion:**

Change in LAS before and after treadmill exercise differentiated Normal SE and abnormal SE (Ischemic SE and Abnormal DST). It may represent a novel non‐invasive assessment for diastolic stress echocardiography.

## Graphical abstract

Left atrial strain is a novel speckle‐tracking technique used in the assessment of left atrial function and has been shown to be a marker for adverse cardiac events. Changes before and after exercise differentiated between normal and abnormal stress echocardiograms, suggesting an additional role in stress echocardiography.

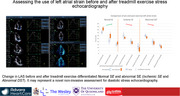

**Abbreviations**
2D‐STE: two‐dimensional speckle tracking echocardiography
A2C: apical two‐chamber
A4C: apical four‐chamber
AUC: area under the curve
BMI: body mass index
BSA: body surface area
DST: diastolic stress test
EF: ejection fraction
HFpEF: heart failure with preserved EF
HFrEF: heart failure with reduced EF
LA: left atrium
LAS: LA strain
METs: metabolic equivalents for task
ROC: receiver operator curve
SE: stress echocardiogram

## 1 Introduction

The assessment of diastolic dysfunction is gaining attention due to the emergence and importance of heart failure with preserved ejection fraction (HFpEF). It is estimated that over 50% of heart failure patients have this form of cardiac dysfunction [1, 2, 3, 4]. The role of the left atrium (LA) in this process is also becoming more apparent. Echocardiography permits a low risk non‐invasive assessment of diastolic parameters, including evaluation of LA and its function [4, 5, 6, 7].

**FIGURE 1:**
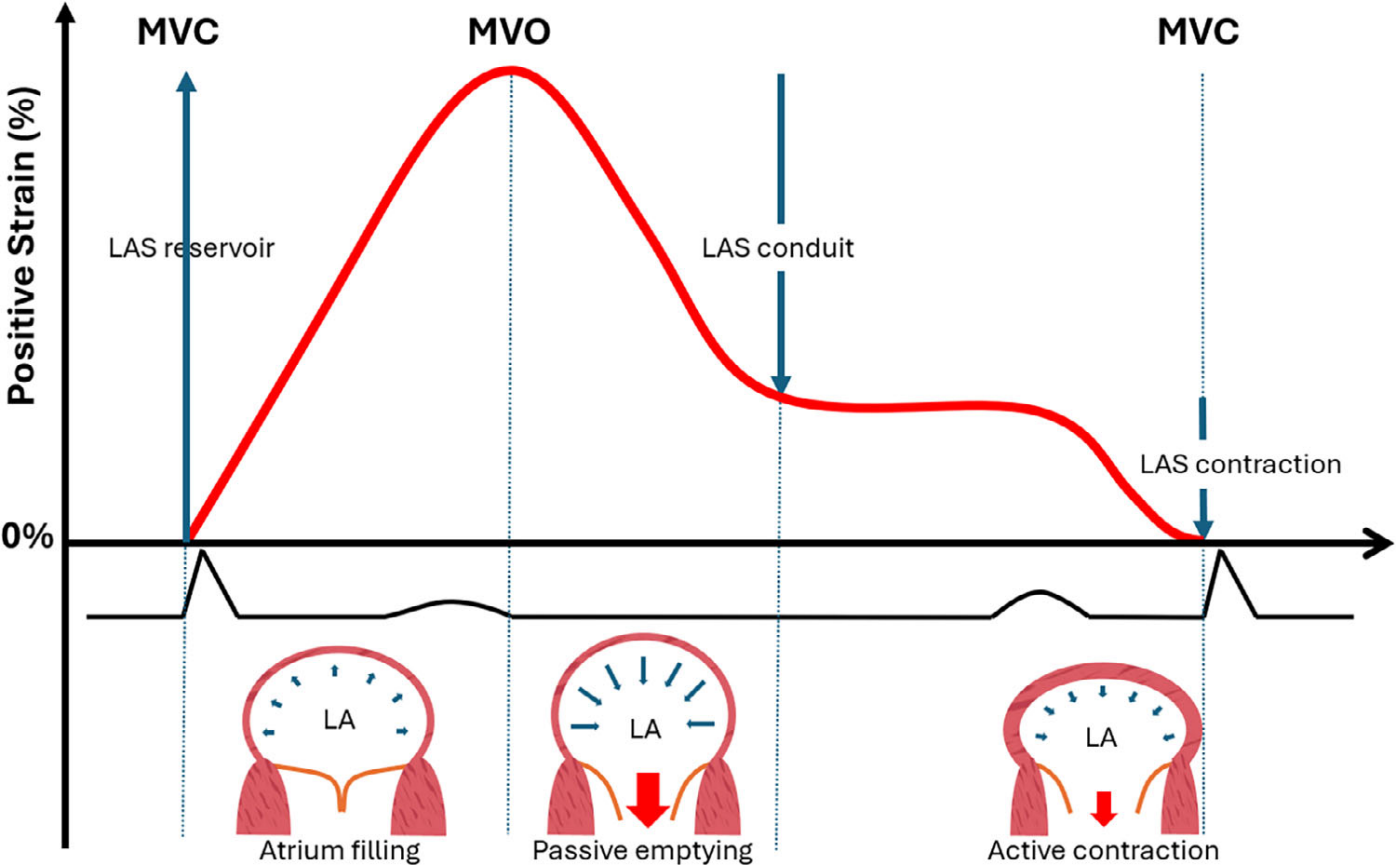
Diagram summarizing the pathophysiology of left atrial strain, with respect to left atrial filling and the ECG. ECG, electrocardiogram; LA, left atrium; LAS, left atrial strain; MVC, mitral valve closure; MVO, mitral valve opening.

The LA has a role in receiving blood from the returning venous circulation (acting as a reservoir). It then permits the passive transfer of blood via a small pressure gradient to the left ventricle (acting as a conduit). During late left ventricular (LV) diastole, it actively pumps blood, adding to LV stroke volume, in its role as a contractile pump. Finally, there is left atrial suction to assist in left atrial early systolic filling (see Figure 1, [5]).

Left atrial strain (LAS) is a novel speckle‐tracking echocardiographic technique that quantifies the magnitude and rate of LA myocardial deformation. It is regarded as being less load dependent compared to the volumetric evaluation of the LA [5, 6, 7]. The technique is now semi‐automated and is less affected by artefact and measurement angle than other echocardiographic methods of measurement [5]. It appears to be a sensitive marker for early diastolic dysfunction and correlates with LV filling pressures [5, 8]. It is an early marker for adverse cardiac events, particularly in heart failure, myocardial ischemia, and atrial fibrillation [5, 8, 9, 10, 11]. The three most commonly used LAS parameters measure reservoir, conduit, and contractile LA function [5, 8, 9, 10, 11].

Exercise stress echocardiography is well established and validated for the evaluation of myocardial ischemia [12]. It is a recommended investigation for the evaluation of diastolic function [4, 13] and has also been shown to have prognostic value following the diastolic stress test (DST) [14, 15]. LV filling pressures may be normal at rest, but significantly increase with exertion [4, 13, 16]. The DST involves measuring echocardiographic parameters for LV filling at rest and after stress [4]. Incorporation of LAS before exercise stress testing has been shown to independently predict exercise capacity in heart failure patients [17, 18]. As a marker of diastolic function, LAS may assist with the determination of diastolic dysfunction and with the assessment of myocardial ischemia. When measured at rest before stress testing, it has been suggested to predict DST results [19, 20]. Its role in undifferentiated patients has not been assessed before *and after* treadmill exercise.

This novel study aims to determine the role and value of LAS in the stress echocardiographic assessment of myocardial ischemia and diastolic function. The concept was to assess if there was a change in LAS before and after treadmill exercise and to assess if these changes differentiated between patients with non‐ischemic SE with normal filling pressures post‐exertion (*Normal SE*) and those patients with *ischemic SE* and abnormal diastolic stress tests (*Abnormal DST*), as defined below.

## 2 Materials and Methods

### 2.1 Study Population

This study was a retrospective analysis of data collected from consecutive patients referred to Advara HeartCare clinical testing facilities for exercise stress echocardiography in Brisbane, Australia, between April 17, 2023 and November 27, 2023. Patients were referred for investigation of chest pain, dyspnea, or assessment of silent ischemia. Standard Bruce protocol treadmill testing [21] with digital gated echocardiography was utilized. Ethics was approved by the UnitingCare Health Human Research Ethics Committee.

### 2.2 Echocardiographic Assessment

The echocardiographic assessment was completed before and after treadmill exercise (regional wall analysis within 90 seconds and additional Doppler assessments within 180 seconds of cessation of exercise). Patients with paced rhythm or atrial fibrillation, ejection fractions (EFs) less than 50%, significant valvular stenosis, regurgitation, or valve surgery (aortic or mitral replacement or mitral repair), or patients requiring dobutamine stress were excluded. Patients with reduced EF were excluded due to the possibility of having increased LA filling pressures at rest. Patients with pre‐existing regional wall motion abnormalities were excluded, as these changes would influence LAS values at rest and potentially post‐exertion.

Echocardiographic images were acquired at rest and after treadmill exercise in the parasternal long‐axis, short‐axis, apical four‐ (A4C), two‐ (A2C), and three‐chamber views. The EF was estimated by Simpson's method of discs. Assessment of LA deformation was performed using the latest General Electric HealthCare EchoPAC (Waukesha, Wisconsin, USA) software from A4C, A2C, and Biplane images, including the LA. Frame rates were recorded between 50 and 70 frames per second (FPS). Modalities utilized included left atrial reservoir, conduit, and contractile strain. These images were recorded before and after exercise. The post‐exertion imaging protocol involved the regional wall motion analysis being performed first. This was completed in 40 to 90 seconds. The tricuspid (TR) velocity was imaged next, taking less than 10 seconds. The A4C LA focused view and A2C focused view were recorded next, taking another 15–20 seconds. Frame rates were recorded between 50 and 70 FPS, similar to or the same as at rest. This meant that LA imaging was routinely recorded between 90 and 120 seconds after the completion of treadmill exercise. The offline analysis for LAS also takes very little time. In the paper by Rausch et al., the analysis time for experts was 91 ± 17 seconds and for novices 156 ± 63 seconds [22]. The mitral inflow and Doppler tissue imaging were analyzed last, typically done at the 120 seconds post‐exercise mark. If the E and A waves had not separated by 120 seconds, then the analysis was delayed until that had occurred.

Two‐dimensional speckle tracking echocardiographic (2D‐STE) derived LAS was automatically generated by the software (with LA endocardial tracking) and manually adjusted where necessary. The pulmonary veins and LA appendage (when visible) were not included for these measurements. The software then calculated the reservoir, conduit, and contractile LA strain, and the A4C, A2C, and biplane measurements were recorded.

The resting and stress echocardiogram was performed by cardiac sonographers with subspecialty training and expertise in stress echocardiography. Exercise was supervised by an exercise physiologist. All tests were supervised and read by cardiologists with subspecialty training in stress echocardiography. Results were then over‐read and standardized by a single stress echocardiography specialized cardiologist who was blinded to the results of the initial tests. This medical doctor then conducted the LAS analysis.

Ischemic tests (*Ischemic SE*) were defined as having regional wall motion abnormalities in two or more contiguous segments, or tests showing cavity dilatation with a lack of cardiac augmentation (based on direct comparison of the pre‐ and post‐exercise echocardiographic images). Abnormal DSTs were defined as having an increase in the medial E/e’ to greater than or equal to 12, without the above ischemic abnormalities. Previous research has shown abnormal results for the DST with a variety of E/e’ values [4, 14, 15]. An E/e’ > 10 has been shown to be a sensitive marker of diastolic dysfunction, while an E/e’ > 15 was more specific [16]. The first paper to show a prognostic change for the DST used a cut point of ≥ 12. A subsequent paper to show prognostic change for the DST revealed abnormal values with a variety of E/e’ values from 10 and above, with the greatest change with E/e’ ≥ 15. On the basis that prognostic change was detected with an E/e’ ≥ 12 in two papers and that an E/e’ ≥ 12 was the cut point for heart failure outcomes, this research selected the above definition [14, 15]. *Normal SE* were defined as having non‐ischemic stress images and the post‐exercise medial E/e’ did not increase to ≥12. A subset of patients went on to have an invasive coronary angiogram or a CT coronary angiogram, determined and organized by the treating cardiologist, independent of the study.

**FIGURE 2:**
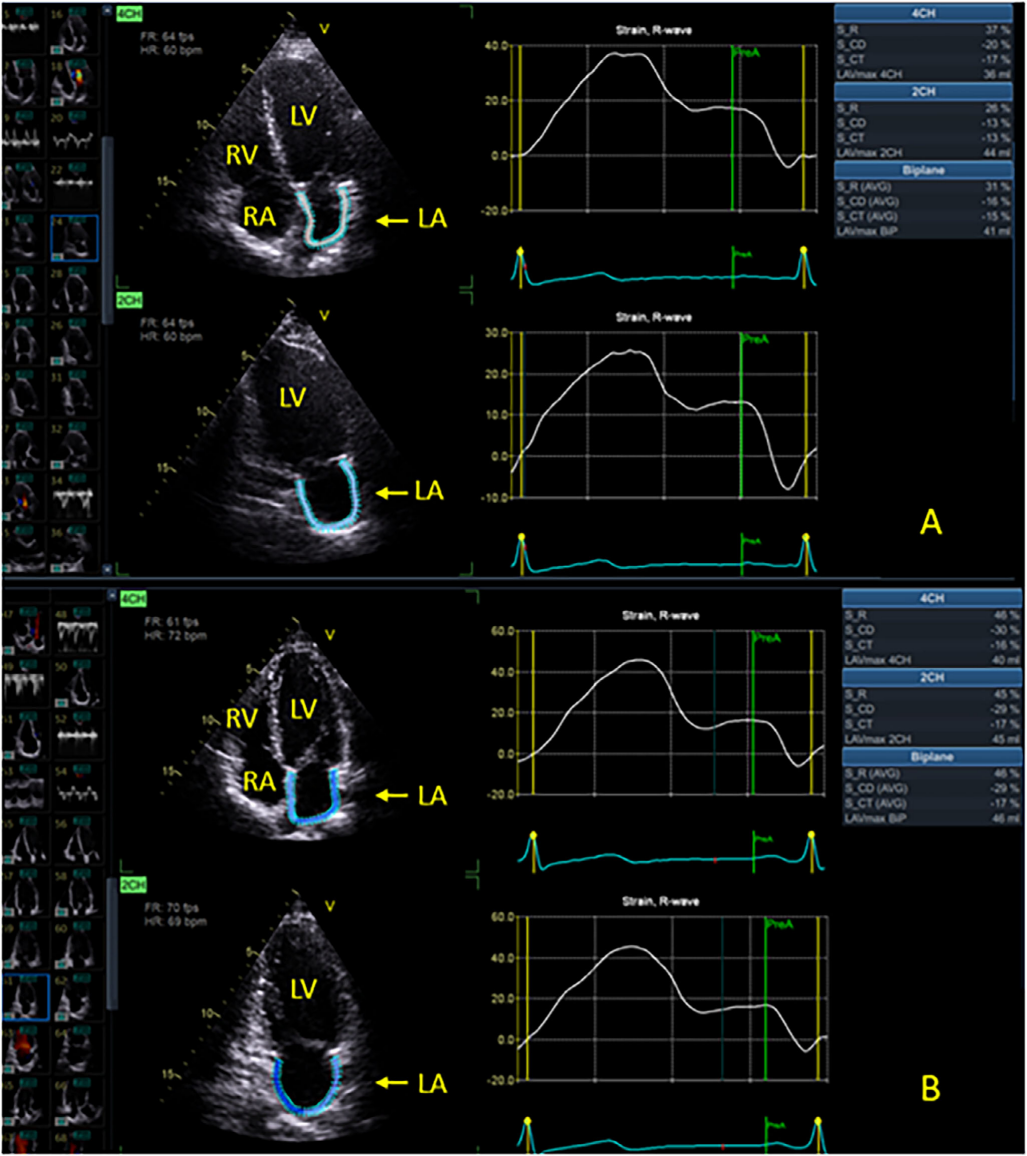
Normal General Electric EchoPAC left atrial strain before and after exercise (*normal SE*). (A) Pre‐exercise LAS in the A4C and A2C. (B) Post‐exercise LAS. A4C, apical four chamber; A2C, apical two chamber; LAS, left atrial strain; SE, stress echocardiogram.

**FIGURE 3:**
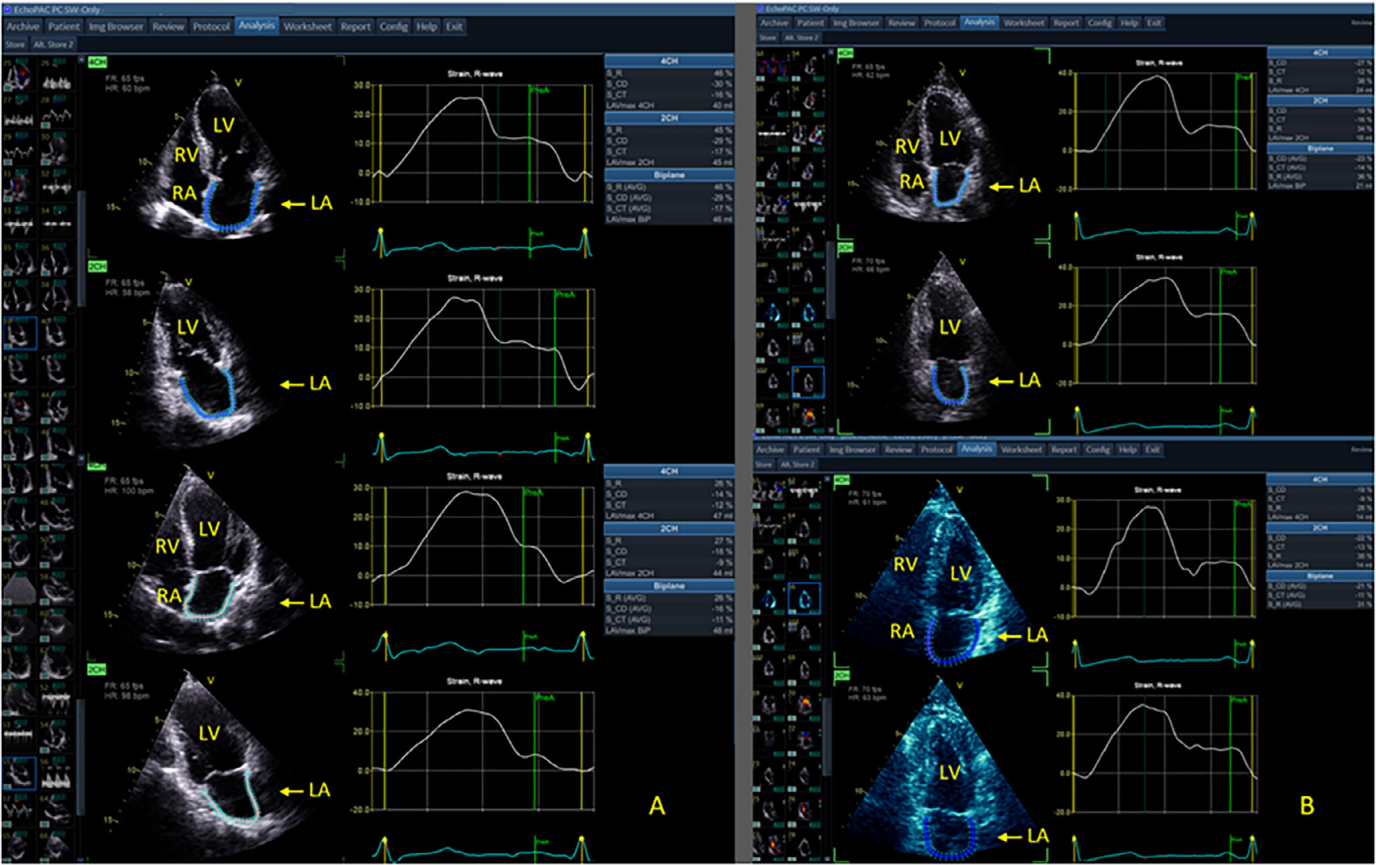
General Electric EchoPAC left atrial strain before and after exercise. (A) Patient with an ischemic stress echocardiogram (Ischemic SE); (B) Patient with a non‐ischemic stress echocardiogram but abnormal diastolic stress test (abnormal DST). DST, diastolic stress test; SE, stress echocardiogram.

In order to assess the value and utility of LAS in stress echocardiography, a post‐exercise strain analysis was conducted. The analysis was performed independently of and blinded to the stress test results. Left atrial reservoir strain, conduit strain, and contractile strain measurements were made on the before and after exercise A4C and A2C images, with the LA emphasized. The R‐wave gating method was employed. General Electric HealthCare EchoPAC (Waukesha, Wisconsin, USA) systems were used to perform the LAS analysis (see Figures 2 and 3).

### 2.3 Exercise Assessment

Exercise was replicated and quantitated using General Electric medical grade treadmills using Case Systems (Milwaukee, Wisconsin, USA). Standard Bruce protocols were utilized to replicate exercise stress in a controlled and reproducible method. Metabolic equivalents for task (METs) were estimated to quantify and compare exercise capacity. Imaging was acquired using General Electric Vivid e95 (Horton, Kansas, USA) ultrasound scanners.

### 2.4 Statistical Analysis

Data were exported in Microsoft Excel (Washington, USA) format for subsequent statistical analysis using Medcalc Statistical Software (Ostend, Belgium). Baseline and demographic data were tabulated and summarized using descriptive statistics. Paired *t*‐tests were used to compare continuous data for dependent variables. Unpaired *t*‐tests were utilized for cohort analysis of continuous data for separate populations. ANOVA, one‐way analysis of variance, was used for testing the differences between multiple groups.

Data expression was presented as the mean plus or minus the standard deviation. A receiver operator curve (ROC) analysis was performed to assess if there was a difference in performance between the types of strain (reservoir, conduit, contractile), and in each echocardiographic area of measurement (A4C, A2C biplane). A multiple linear regression analysis was conducted to assess whether LAS was predictive of change.

For the analysis, patients were grouped by the result from the stress echocardiogram (SE). There were three (3) groups (as defined above): patients with a *Normal SE*; patients with an ischemic SE (as defined above), called *Ischemic SE*; and patients with a non‐ischemic SE but abnormal DST (as defined above), called *Abnormal DST*. The LAS values were measured and analyzed before and after exercise and subsequently compared. No funding was required for this research.

## 3 Results

### 3.1 Patient Population

There were 457 consecutive patients referred for treadmill exercise stress echocardiography during the investigation period. Of these, 37 had pre‐determined exclusions and were removed from the analysis (5 with pacemakers, 20 with atrial fibrillation, 6 with EF less than 50%, and 11 with valvular heart disease). The remaining 420 stress echocardiograms (with 155 female patients, making up 37% of the total) were available for analysis. The mean age of this group was 61.4 ± 12.6 years. The mean body mass index (BMI) was 28.2 ± 5.1 kg/m^2^, and the mean body surface area (BSA) was 1.97 ± 0.22 m^2^. The mean EF was 62% ± 5% and the mean left atrial volume was 33.0 ± 9.3 mL/m^2^. Patients exercised for a mean of 11.1 ± 3.4 METs. The mean resting heart rate was 69 ± 11 bpm, with a mean resting blood pressure of 133 ± 16 mmHg (see Table 1). From this group, there were 367 with complete pre‐ and post‐exercise data (87% of all stress echocardiograms). This included 313 *Normal SE*. There were 32 *Ischemic SE*, 29 of which had a subsequent invasive coronary angiogram. Severe coronary artery disease (≥70% luminal stenosis) was seen in 24 of these 29 studies, resulting in an acceptable 83% true positive rate for obstructive coronary disease. The mean wall motion score for this group was 1.6 ± 0.4. There were another 22 tests that had an *Abnormal DST* (non‐ischemic stress images but E/e’ post exertion ≥12). The baseline characteristics for the groups are listed in Table 1. The HR and BP before and after exercise for each of the groups are compared in Table 1. There were significantly more female patients in the *Abnormal DST* group compared to the *Normal SE* group. The BSA and exercise capacity were lower in the *Abnormal DST* group compared to the *Normal SE* cohort. Some of this difference may be explained by there being more females in this group. The *Ischemic SE* patients had statistically lower EF (but still within the normal range) and exercise capacity (see Table 1).

**TABLE 1:** Baseline characteristics comparing the total group (*n* = 420), normal SE (*n* = 313), ischemic SE (*n* = 32), and abnormal DST (*n* = 22).

	All Patients	Normal SE	Ischaemic SE	Abnormal DST	*p* value	95% CI
**Mean Age**	61.4 ± 12.6					
**Females**	37% (n=155)	36%	28%	**64%**	**0.009**	**0.1 to 0.5**
**Mean BMI (kg/m^2^)**	28.2 ± 5.1	28 ± 5	28 ± 5	29 ± 6		
**Mean BSA (m^2^)**	1.97 ± 0.22	1.97 ± 0.23	1.97 ± 0.18	1.89 ± 0.29		
**Comorbidities**						
**IHD**	187 (52%)	156 (50%)	**23 (72%)**		**0.02**	**0.4‐4.0**
				9 (41%)		
**HT**	139 (38%)	107 (34%)	**19 (59%)**		**0.005**	**0.08‐0.43**
				**12 (55%)**	**0.054**	**−0.03 to 0.41**
**DM**	45 (12%)	36 (12%)	4 (13%)			
				3 (14%)		
**AF**	18 (5%)	14 (5%)	2 (6%)	2 (9%)		
**VHD**	11 (3%)	9 (3%)	1 (3%)	0		
**Mean EF**	62% ± 5%	62 ± 5	**60 ± 5**		**0.001**	**−4.4 to −1.1**
				60 ± 5		
**Mean LAV (ml/m^2^)**	33.0 ± 9.3	32.7 ± 9.3	35.0 ± 8.5	35.0 ± 2.5		
**E/e’ rest**	9.9 ± 3.0	9.8 ± 3.0	10.4 ± 3.1	10.6 ± 3.3		
**E/e’ post**	9.3 ± 2.9	8.6 ± 2.2	12.3 ± 3.6	15.3 ± 3.3		
**TR velocity rest**	2.4 ± 0.3	2.4 ± 1.3	2.5 ± 0.3	2.5 ± 0.2		
**TR velocity post**	3.0 ± 1.8	3.0 ± 2.0	3.0 ± 0.4	3.1 ± 0.7		
**Mean Exercise capacity** in METs	11.1 ± 3.4	11.4 ± 3.3	**9.6 ± 2.9**		**0.002**	**−3.0 to −0.7**
				**7.9 ± 3.4**	**<0.001**	**−5.0 to −2.1**
**Mean HR at rest** (bpm)	69 ± 11	69 ± 11	68 ± 9	71 ± 13		
**Mean HR post** (bpm)	155 ± 17	155 ± 18	151 ± 14	**144 ± 18**	**0.001**	**−19 to −3**
**Mean BP at rest** (mmHg)	132 ±16	132 ± 16	139 ± 17	137 ± 15		
**Mean BP post** (mmHg)	177 ± 21	176 ± 22	183 ± 20	178 ± 22		

*Note*: *p* value reflects levels of significance between relevant patient subgroups with the overall test population.

Abbreviations: AF, atrial fibrillation; BP, blood pressure; BMI, body mass index; BSA, body surface area; DM, diabetes mellitus; EF, ejection fraction; HR, heart rate; HT, hypertension; IHD, ischemic heart disease; LAV, left atrial volume; METs, metabolic equivalent for task; SD, standard deviation; VHD, valvular heart disease.

There were 53 patients (13% of the total) who did not have pre‐ and post‐data available. All of these had image quality that prevented appropriate LAS tracking and so could not be analyzed.

### 3.2 Left Atrial Strain Analysis

In the *Normal SE* cohort, all LAS parameters (reservoir, conduit, and contractile strain in the A4C, A2C, and biplane measurements) increased in a statistically significant manner in the post‐exercise images, compared to the resting images (see Table S1 and Figure 2). This finding suggests that *Normal SE* has LAS contractile reserve from rest to post‐exercise. This increase in LAS post‐exercise in *Normal SE* was seen for each type of strain. As a serial comparison during stress, contractile reserve represents the absolute percentage increase in strain from rest to post‐exercise.

In patients with an *Ischemic SE*, LAS did not statistically significantly change, except for LA reservoir A4C strain and contractile strain (A4C, A2C, and biplane), where LAS statistically decreased post‐exercise, compared to the resting values (see Table S2 and Figure 3). These limited examples of change were the opposite of what occurred in *Normal SE*.

For patients who had an *Abnormal DST*, LAS did not significantly change after exertion, compared to the pre‐exercise imaging, except for the reservoir LAS (A2C and biplane measurements) and contractile LAS (A4C, Biplane), where post‐exercise values decreased statistically compared to the resting images (see Table S3 and Figure 3). Again, this was the opposite of what occurred in *Normal SE*.

Patients in the *Ischemic SE* and *Abnormal DST* groups appeared to have numerically lower LAS at rest compared to the *Normal SE* group. The mean values, however, were not statistically different.

**FIGURE 4:**
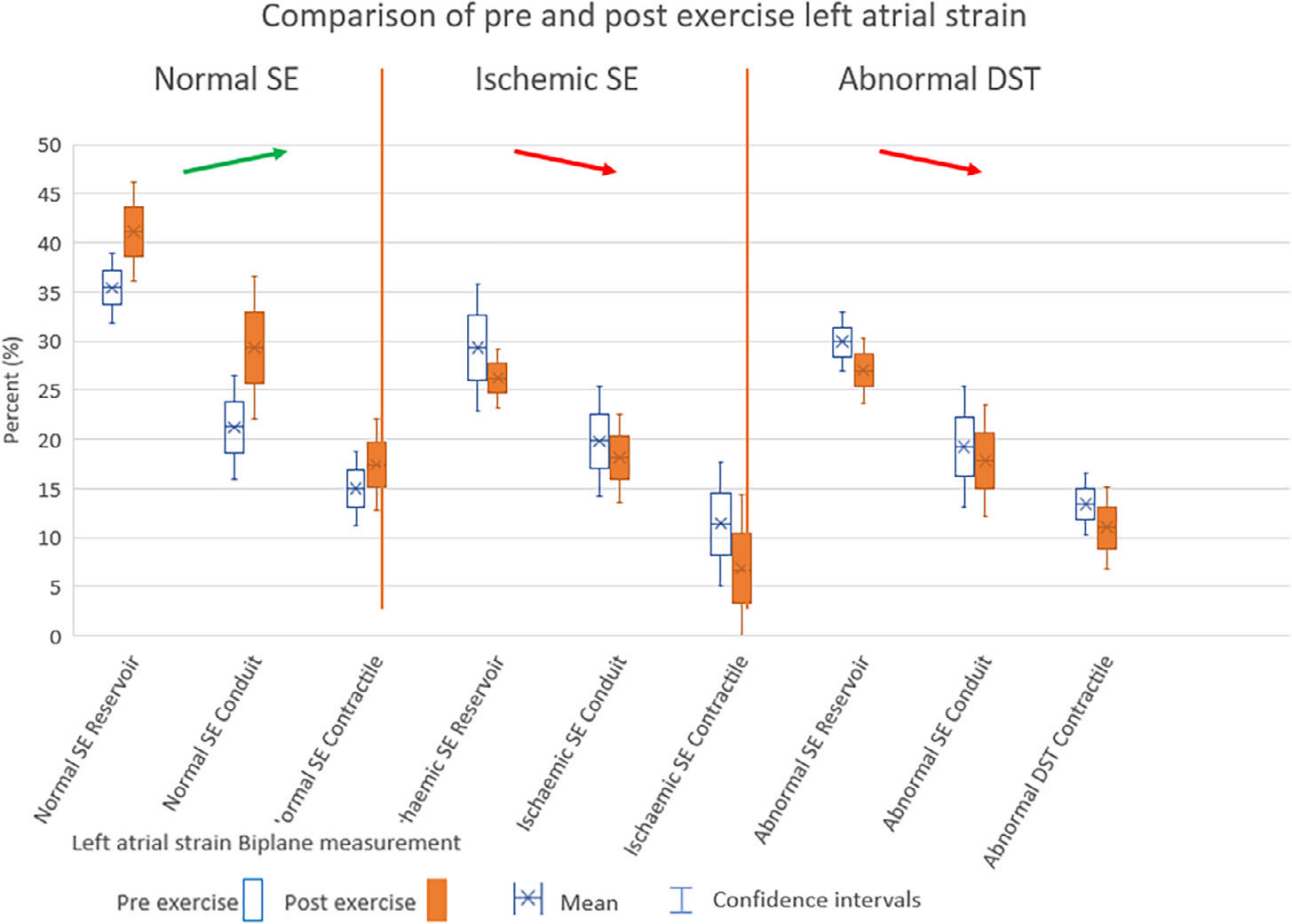
Visual comparison of pre‐ and post‐exercise left atrial strain. Left atrial strain is expressed as an absolute percentage to simplify its visual representation. DST, diastolic stress test; SE, stress echocardiogram.

This change between rest and post‐exertion LAS values for *Normal SE* and the difference in the *Ischemic SE* post and pre‐exercise LAS measurements were compared. The differences (an increase in LAS in *Normal SE* and no change or small decrease in LAS for *Ischemic SE*) were shown to be statistically different (see Table 2). The *Normal SE* LAS change with exercise was also statistically different compared to the *Abnormal DST* LAS change with exercise (no change or small decrease in LAS) (see Table 2). However, the difference in LAS post versus pre‐exercise for *Ischemic SE* and *Abnormal DST* was not statistically different (see Table 3 and Figure 4). The ANOVA analysis also did not suggest that there were differences between the abnormal SE groups.

**TABLE 2:** Comparison of the difference in LA Strain post versus pre‐exercise for *normal SE* versus *ischemic SE* and *abnormal DST*.

Left atrial strain	Patient subgroup	Difference post‐exercise versus pre‐exercise	*p* value
A4C reservoir	Normal SE	7.6 ± 4.5	
	Ischemic SE	−4.6 ± 5.9	<0.001
	Abnormal DST	−2.4 ± 5.9	<0.001
	Ischemic + Abnormal DST	−3.7 ± 5.9	<0.001
A4C conduit	Normal SE	−8.8 ± 5.8	
	Ischemic SE	1.3 ± 8.4	<0.001
	Abnormal DST	1.2 ± 5.2	<0.001
	Ischemic + Abnormal DST	1.3 ± 7.2	<0.001
A4C contractile	Normal SE	−2.4 ± 3.4	
	Ischemic SE	2.4 ± 2.5	<0.001
	Abnormal DST	3.2 ± 4.9	<0.001
	Ischemic + Abnormal DST	2.7 ± 3.6	<0.001
A2C reservoir	Normal SE	7.1 ± 4.6	
	Ischemic SE	−5.4 ± 5.2	<0.001
	Abnormal DST	−3.3 ± 5.6	<0.001
	Ischemic + Abnormal DST	−4.6 ± 5.4	<0.001
A2C conduit	Normal SE	−7.8 ± 6.5	
	Ischemic SE	1.5 ± 6.0	<0.001
	Abnormal DST	1.9 ± 7.5	<0.001
	Ischemic + Abnormal DST	1.7 ± 6.6	<0.001
A2C contractile	Normal SE	−2.5 ± 3.9	
	Ischemic SE	4.5 ± 3.7	<0.001
	Abnormal DST	1.6 ± 3.6	<0.001
	Ischemic + Abnormal DST	3.4 ± 3.9	<0.001
Biplane reservoir	Normal SE	7.4 ± 5.8	
	Ischemic SE	−5.0 ± 5.1	<0.001
	Abnormal DST	−2.9 ± 5.1	<0.001
	Ischemic + Abnormal DST	−4.2 ± 5.2	<0.001
Biplane conduit	Normal SE	−8.2 ± 5.2	
	Ischemic SE	1.8 ± 6.3	<0.001
	Abnormal DST	1.4 ± 5.4	<0.001
	Ischemic + Abnormal DST	1.6 ± 5.9	<0.001
Biplane contractile	Normal SE	−2.4 ± 2.9	
	Ischemic SE	3.3 ± 2.4	<0.001
	Abnormal DST	2.6 ± 3.7	<0.001
	Ischemic + Abnormal DST	3.1 ± 3.0	<0.001

*Note: ANOVA, one‐way analysis of variance for testing of differences between the three groups (Normal SE, Ischemic SE, and Abnormal DST)*

Unpaired t tests were utilized for analysis of the continuous data comparing *Normal SE* and the combined abnormal tests.

For all strain measurements, the Scheffe test for pairwise comparisons was not different for the *Ischemic* and *Abnormal DST* groups (p>0.05).

For all comparisons, the D'Agostino‐Pearson test rejected normality between Normal SE LAS measurements and those for abnormal tests (*Ischemic* and *Abnormal DST*).

Abbreviations: A4C, apical four chamber; A2C, apical two chamber; CI, confidence interval; DST, diastolic stress test; LAS, left atrial strain; SE, stress echocardiogram

**TABLE 3:** Comparison of the difference in LAS post versus pre‐exercise between *ischemic SE* and *abnormal DST*.

Test	Ischemic SE	Abnormal DST	Difference	*p* value
A4C reservoir	−4.6 ± 5.9	−4.2 ± 10.4	0.4	0.87
A4C conduit	1.3 ± 8.4	1.2 ± 5.2	−0.1	0.96
A4C contractile	2.4 ± 2.5	3.2 ± 4.9	0.8	0.52
A2C reservoir	−5.4 ± 5.2	−3.33 ± 5.6	2.0	0.19
A2C conduit	1.5 ± 6.0	1.9 ± 7.5	0.4	0.83
A2C contractile	4.5 ± 3.7	1.6 ± 3.6	−2.9	0.07
Biplane reservoir	−5.0 ± 5.1	−2.9 ± 5.1	2.2	0.14
Biplane conduit	1.8 ± 6.3	1.4 ± 5.4	−0.3	0.83
Biplane contractile	3.3 ± 2.4	2.6 ± 3.7	−0.7	0.43

Abbreviations: A4C, apical four chamber; A2C, apical two chamber; DST, diastolic stress test; SE, stress echocardiogram.

A ROC analysis was performed to evaluate how the individual parameters performed for patients with an *Ischemic SE* and an *Abnormal DST*. The best results were seen with Biplane Reservoir strain. For this parameter, the AUC for patients with an *Ischemic SE* was 0.813, with a sensitivity of 98% and a specificity of 64% (*p* < 0.0001). For the patients with an *Abnormal DST*, the AUC was 0.759 with a sensitivity of 99% and a specificity of 53% (*p* < 0.001). The best single measurement was the A4C Reservoir strain, with an AUC for *Ischemic SE* of 0.802, with a sensitivity of 98% and a specificity of 63% (*p* < 0.001), and for *Abnormal DST*, an AUC of 0.743 with a sensitivity of 97% and a specificity of 54% (*p *< 0.001). In general, the LAS parameters were highly sensitive, with lower specificity.

Change in LAS was then assessed using a multiple linear regression analysis to evaluate its adjunctive value to stress echocardiography. The independent variables assessed included E/e’, TR velocity, METs, Biplane reservoir LAS, and A4C LAS (these two strain parameters were chosen based on the ROC analysis). The E/e’ and A4C Reservoir strain were found to be predictive of an *Ischemic SE* with an adjusted *r* ^2^ of 0.48 (see Table S4). For patients with an *Abnormal DST*, numbers were insufficient for a meaningful analysis. When the results of the *Ischemic SE* were combined with those of the *Abnormal DST* (i.e., all patients with abnormal diastology post exercise), similar results emerged, with E/e’ and A4C Reservoir strain predictive of the combined dependent variable (*r* ^2^ = 0.57, see Table S4).

## 4 Discussion

Assessment of the LA is gaining recognition due to its role in left heart filling, diastolic function, and as a marker of adverse cardiac outcomes. LAS is an emerging technique that appears relatively independent of tethering and load effects. It has been shown to be predictive of adverse events [5, 9, 10]. Speckle tracking LAS has become the preferred technique as it is semi‐automated, less angle dependent, and less prone to artifacts compared to other modalities. It is quick to use and learn, and is reproducible [5, 22, 23].

Strain is load‐dependent. The higher the heart rate, the greater the change in LAS and the greater the peak LAS. Higher heart rates result in reduced ventricular filling times. Decreased ventricular afterload, which enhances forward flow (i.e., ventricular ejection), reduces end‐systolic volume and end‐diastolic volume secondarily. This principle is likely to apply to the LA [5]. There is significant variation in LAS values for individuals as represented by the wider standard deviations of the means (see the Tables in Supporting Information).

LAS assessment permits differentiation between active myocardial deformation and passive wall motion. The LAS parameters independently correlate with markers of LV systolic and diastolic function, such as stroke volume and E, A, and e’ velocities. Studies have shown that using LAS added prognostic value and was predictive for the detection of HFpEF [5, 7, 9]. Other research has suggested that accuracy increased when LAS measurements were used in addition to the diastolic function guidelines algorithm [4, 6].

Why would LAS be different in patients with *Abnormal DST* and *Ischemic SE* compared to patients with *Normal SE*? Patients with a normal response are presumed to have normal filling pressures with exertion. In *Normal SE*, LAS parameters were shown to increase, corresponding to an expected improvement in LV and LA filling. In patients with *Abnormal DST*, the LA and LV filling pressures are presumed to increase, due to a less compliant LV, resulting in an abnormal LAS response. For patients with an *Ischemic SE*, myocardial ischemia in itself produces diastolic dysfunction [4, 24]. In the ischemic cascade, abnormalities of myocardial relaxation occur early [25]. Additionally, contractile change and regional wall motion abnormalities are likely to impair LV filling with potential additional effects on LAS [19, 26].

This study showed that assessment of LAS before and after treadmill exercise is feasible, quick, and readily performed. Results can be obtained in the vast majority of patients. The current standard parameters of LAS (reservoir, conduit, and contractile strain) statistically increase post‐stress testing in normal patients (those without ischemia or other evidence of diastolic dysfunction) (see Figure 2). In contrast to this, patients with evidence of myocardial ischemia did not have a statistical increase in LAS markers (see Figure 3). A similar pattern (no statistically significant increase in LAS) was seen in patients with an abnormal DST (see Figure 3). LAS, therefore, appears to be able to discriminate readily between normal and abnormal tests. The differing results in LAS with exercise may be another marker of an abnormal diastolic function response. A change in LA filling pressures and LA filling may result in an abnormal LAS response post‐exertion. It may also be a result of inherent LA dysfunction. The mechanisms are postulated and yet to be proven.

Patients in the *Ischemic SE* and *Abnormal DST* groups appeared to have numerically lower LAS at rest compared to the *Normal SE* group. It may be that the baseline values are lower because the ischemic and DST groups exhibit subclinical atrial dysfunction even at baseline (before exercise). This observation would support LAS being a very sensitive technique to detect early atrial dysfunction. However, these apparent numerical disparities were not statistically different.

Previous research had suggested that baseline LAS could be discriminatory [27]. There are however questions regarding the cutoff values. The value of performing the LAS analysis post‐exertion would be to confirm the abnormal response and to ensure low or borderline pretest results are truly abnormal. For an individual presenting for testing, an initial low or borderline value may be difficult to differentiate from a Normal SE whose pre‐stress LAS is at the lower end of the normal range. The post‐test result confirms whether there is a normal or abnormal response to exercise.

Previous research has also shown that lower LAS values before diastolic stress testing were predictive of an abnormal DST, even more so than pre‐test E/e’, indexed LA volume and pulmonary arterial systolic pressures [27].

Sugimoto et al. assessed reservoir LAS before, during and after cardiopulmonary exercise testing, in healthy controls, and in patients with reduced EF heart failure (HFrEF) and HFpEF. Normal subjects had an increase in LAS (as seen in in our *Normal SE* group) and HFrEF patients had no change in LAS. Patients with HFpEF had an increase in LAS, different to what was seen in our cohort [26]. It may be that in that study the patients with HFpEF had been treated, lowering baseline LA pressures, with a corresponding normalization of the LAS exercise response. Our research was in patients where the diagnosis was unknown before testing. Patients were therefore not on treatment for diastolic dysfunction. These data showed that LAS is abnormal (does not increase) in untreated patients with an abnormal DST. It suggests that the test can be used for all patients presenting with shortness of breath. While it can be used in a targeted fashion (in the DST), it can be analysed for all patients being sent for stress echocardiography.

The data presented here do not readily lead to a cutoff value for LAS. The problem with providing a cutoff is that there is significant variation in baseline values between individuals. What this paper suggests is that patients with *Normal SE* will have an increase in the value of strain from rest to peak, whereas patients with abnormal SE (both *Ischemic SE* and *Abnormal DST*) do not increase their baseline LAS. The lack of change is a significant abnormality.

The role of the estimation of diastolic dysfunction is now established in guidelines, which describe the DST and recommend its use in appropriate circumstances [4]. The use of a quick and reproducible technique such as LAS [23] is intriguing, with the potential to add to the current diagnostic algorithm for diastolic function assessment. It is quick and easy to do and only requires two extra images to be recorded before and after stress testing. Due to these factors, LAS could easily be added to the DST, as a functional marker. The prognostic value of this role would need further investigation.

There were some differences between groups at baseline. Significantly more females were present in the *Abnormal DST* group, compared to the *Normal SE* group. In HFpEF, female patients are over‐represented compared to other cardiac diagnoses, so this finding was to be expected [28]. Females who had *Normal SE* had an appropriate increase in LAS with exertion, similar to the overall result, so having more females in the *Abnormal DST* group should not have influenced these test results. Females have lower BSA compared to males (21), explaining why BSA was lower in the baseline characteristics for that group of patients. Exercise capacity was lower in both the *Ischemic SE* group and the *Abnormal DST* group. Both of those pathologies would be expected to result in reduced exercise capacity compared to patients with *Normal SE*. Patients with lower exercise capacity in the *Normal SE* group still had an appropriate increment in LAS post‐exertion, similar to the overall *Normal SE* patients. Therefore, it was unlikely that the baseline differences contributed significantly to the results.

### 4.1 Study Limitations

There are limitations in this study. This was a non‐randomized, single center, observational cohort, analyzed retrospectively. It was a heterogeneous group, with indications for the test including chest pain, shortness of breath, assessment of known coronary artery disease, and for the assessment of patients with increased cardiovascular risk. With respect to the assessment of diastolic function, patients were not exclusively referred for this (although it was one of the referral indications). Nonetheless, this is reflective of patient populations being sent for routine stress testing and is therefore applicable in that setting. It would extend the utility of LAS testing to a general stress echocardiographic population. These findings should be retested separately in an independent patient population for confirmation.

The majority of *Ischemic SE* patients had an anatomical test to confirm the diagnosis. Ideally, all patients would have their anatomy documented to confirm the presence or absence of significant coronary disease, as well as measuring diastolic parameters invasively, in a blinded fashion, after the stress test. This was not regarded as being feasible and would negate the non‐invasive advantage of stress echocardiography. Low to intermediate risk patients (as seen in this cohort) are not recommended to undergo invasive procedures, raising ethical considerations if this were to be suggested. In this scenario, SE is used to avoid an invasive test. Coronary artery computer tomography scanning could have been done and would answer the anatomical question, but with a significant increase in expense and some increased risk. Physicians commonly use one technique or the other to answer this clinical question.

LAS assessment cannot be performed in all patients. Image quality remains the bane of speckle tracking analysis. Full pre‐ and post‐exercise data were available in 87% of studies, however, suggesting that it is feasible for the vast majority of patients. There were fewer females than males, potentially reducing the applicability to women. This is a common finding in research and reflects real‐world experience [14, 28]. Whilst attempts were made to blind the reviewer to the test results, ascertainment and other biases were possible. Validation of this model in an independent cohort with differing ethnic groups would be beneficial and ideal.

## 5 Conclusion

LAS is an emerging technique that is quick and easy to perform. It can be readily assessed before and after treadmill exercise in the majority of patients. The data presented here suggest that LAS assessment may differentiate between normal and ischemic stress echocardiograms. It therefore potentially adds value to the evaluation of myocardial ischemia. Additionally, LAS assessment differentiated between normal and abnormal DSTs and could easily be added to the DST protocol. These data suggest that LAS may be a very useful non‐invasive tool to add to modern stress echocardiography.

## Supporting information




**Supporting File 1**: echo70253‐sup‐0001‐SuppMat.docx.
