# Supplementary material for: Assessing the Use of Left Atrial Strain Before and After Treadmill Exercise Stress Echocardiography
Source: Echocardiography. 2025 Aug 1;42(8):e70253. doi: 10.1111/echo.70253 (PMC12315482; doi:10.1111/echo.70253)
Supplement: Supplementary file 1 — Supporting File 1: echo70253‐sup‐0001‐SuppMat.docx. [file ECHO-42-e70253-s001.docx]

Supplementary Table 1. Comparison of *Normal SE* left atrial strain Pre and Post exercise.

| **Left atrial strain** | **Pre-exercise** | **Post exercise** | **p value** | **95% CI of the difference** |
| --- | --- | --- | --- | --- |
| **Reservoir** |  |  |  |  |
| **A4C** | 36.9 ± 6.9 | 41.8 ± 10.0 | < 0.0001 | 3.8-6.0 |
| **A2C** | 34.4 ± 7.2 | 40.9 ± 12.4 | < 0.0001 | 5.9-7.5 |
| **Biplane** | 35.4 ± 7.0 | 41.1 ± 9.9 | < 0.0001 | 5.4-7.7 |
| **Conduit** |  |  |  |  |
| **A4C** | -21.4 ± 5.9 | -30.2 ± 7.2 | < 0.0001 | -9.3 to -8.1 |
| **A2C** | -21.7 ± 6.0 | 28.9 ± 6.9 | < 0.0001 | -8.5 to -7.1 |
| **Biplane** | -21.2 ± 5.3 | -29.3 ± 7.2 | < 0.0001 | -8.8 to -7.6 |
| **Contractile** |  |  |  |  |
| **A4C** | -14.7 ± 4.5 | -17.2 ± 5.1 | < 0.0001 | -2.8 to -2.0 |
| **A2C** | -15.1 ± 4.4 | -17.6 ± 5.1 | < 0.0001 | -3.0 to -2.0 |
| **Biplane** | -15.0 ± 3.8 | -17.4 ± 4.6 | < 0.0001 | -2.8 to -2.1 |

A4C – apical four chamber; A2C – apical two chamber; CI – confidence interval

Supplementary Table 2. Comparison of *Ischemic SE* left atrial strain pre and post exercise.

| **Left atrial strain** | **Pre-exercise** | **Post exercise** | **p value** | **95% CI of the difference** |
| --- | --- | --- | --- | --- |
| **Reservoir** |  |  |  |  |
| **A4C** | 32.7 ± 4.7 | 28.0 ± 6.9 | 0.0001 | -6.7 to -2.5 |
| **A2C** | 28.8 ± 13.2 | 25.7 ± 6.7 | 0.19 |  |
| **Biplane** | 29.3 ± 13.1 | 26.2 ± 5.9 | 0.25 |  |
| **Conduit** |  |  |  |  |
| **A4C** | -20.9 ± 6.8 | -19.5 ± 5.5 | 0.37 |  |
| **A2C** | -18.8 ± 5.7 | -17.3 ± 4.5 | 0.17 |  |
| **Biplane** | -19.8 ± 5.6 | -18.1 ± 4.5 | 0.12 |  |
| **Contractile** |  |  |  |  |
| **A4C** | -10.1 ± 6.3 | -6.0 ± 8.0 | 0.0002 | 2.1-6.1 |
| **A2C** | -12.5 ± 6.8 | -6.8 ± 7.9 | <0.0001 | 3.3-8.2 |
| **Biplane** | -11.4 ± 6.3 | -6.6 ± 7.7 | 0.0001 | 2.7-6.9 |

A4C – apical four chamber; A2C – apical two chamber; CI – confidence interval

Supplementary Table 3. Comparison *Abnormal DST* left atrial strain pre and post exercise.

| **Left atrial strain** | **Pre-exercise** | **Post exercise** | **p value** | **95% CI of the difference** |
| --- | --- | --- | --- | --- |
| **Reservoir** |  |  |  |  |
| **A4C** | 30.8 ± 6.2 | 28.4 ± 7.0 | 0.08 |  |
| **A2C** | 28.7 ± 6.2 | 25.4 ± 7.1 | 0.01 | -5.9 to -0.8 |
| **Biplane** | 29.9 ± 6.0 | 27.0 ± 6.6 | 0.02 | -5.2 to -0.5 |
| **Conduit** |  |  |  |  |
| **A4C** | -20.5 ± 6.5 | -19.3 ± 5.8 | 0.29 |  |
| **A2C** | -18.1 ± 7.2 | -16.2 ± 6.4 | 0.26 |  |
| **Biplane** | -19.2 ± 6.1 | -17.8 ± 5.7 | 0.24 |  |
| **Contractile** |  |  |  |  |
| **A4C** | -13.4 ± 4.1 | -10.2 ± 4.1 | 0.008 | 0.9 to 5.4 |
| **A2C** | -13.4 ± 3.2 | -11.8 ± 5.3 | 0.06 |  |
| **Biplane** | -13.4 ±3.1 | -11.0 ± 4.2 | 0.004 | 0.9 to 4.3 |

A4C – apical four chamber; A2C – apical two chamber; CI – confidence interval

Supplementary Table 4. Multiple linear regression for LAS

| **Dependent variable** | **Independent variable** | **Coefficient** | **95% CI** | **p value** |
| --- | --- | --- | --- | --- |
| Ischemic SE | E/e’ | 0.06 | 0.03 to 0.09 | 0.0002 |
|  | LAS A4C Reservoir | -0.03 | -0.04 to -0.02 | <0.0001 |
| Ischemic SE + Abnormal DST | E/e’ | 0.08 | 0.05 to 0.11 | <0.0001 |
|  | LAS A4C Reservoir | -0.03 | -0.04 to -0.02 | <0.0001 |

A4C – apical four chamber; CI – confidence interval; DST – diastolic stress test; SE – stress echo
